# Hepatitis B virus: molecular genotypes and HBeAg serological status among HBV-infected patients in the southeast of Brazil

**DOI:** 10.1186/1471-2334-9-149

**Published:** 2009-09-08

**Authors:** Priscila A Tonetto, Neiva SL Gonçales, Viviane C Fais, Aline G Vigani, Eduardo SL Gonçales, Adriana Feltrin, Fernando L Gonçales

**Affiliations:** 1 Grupo de Estudo das Hepatites - Disciplina de Moléstias Infecciosas, Departamento de Clínica Médica - Faculdade de Ciências Médicas - Universidade Estadual de Campinas - UNICAMP; São Paulo, Brazil; 2 Centro de Hematologia e Hemoterapia - Universidade Estadual de Campinas -UNICAMP, São Paulo, Brazil

## Abstract

**Background:**

Knowledge of HBV genotype is very important for clinical treatment. Studies have suggested possible pathogenic and therapeutic differences among HBV genotypes. The aim of this study was to determine HBV subtypes and genotypes in HBV-infected patients in our region (southeast Brazil) and to correlate results with clinical and histopathological data.

**Methods:**

One hundred and thirty-nine HBsAg-positive patients were included in the study. All patients were anti-HCV and anti-HIV negative (64% male; mean age 42 ± 14.5 years; range 7-80 years; 84% Caucasian) and were followed up at the University Hospital. A method for genotyping and subtyping HBV by partial HBsAg gene sequencing with primers common to all known genotypes was used. The viral load was measured by Amplicor Monitor assay (Roche).

**Results:**

HBV genotype A was the most prevalent (55%), while genotypes C, D and F were found in 3%, 38% and 4% of HBV-infected patients, respectively. Among the patients infected by genotype A, 18.3% (14/76) were African descendents and, among the patients infected by genotype D, 11.3% (6/53) were also African descendents. In the four patients infected with genotype C, 2 were Asian descendents and 2 were Caucasians. All (7) genotype F infected patients were Caucasians. Seventy percent of our HBsAg-positive patients were HBeAg negative (62% genotypes A; 26.2% D; 7.1% C and 4.7%F). The viral load of HBV-DNA was about 5 times higher in HBeAg-positive than in HBeAg-negative patients. About 40% of these patients had alanine aminotransferase of up to 1.5 times the normal level. The mean stage of fibrosis in genotype A patients (2.8) was significantly higher than the mean stage of fibrosis in genotype D patients (2.0) (P = 0.0179).

**Conclusion:**

The genotypes encountered in our HBV-infected patients were apparently a consequence of the types of immigration that occurred in our region, where European and African descendents predominate. The HBeAg-negative status predominated, possibly due to the length of time of infection. The viral load in HBeAg-positive patients was higher than in HBeAg-negative individuals. The fibrosis grade in genotype A-infected patients was more advanced than genotype D-infected patients.

## Background

Hepatitis B virus (HBV) infection is endemic in many parts of the world. More than 2 billion people are infected by HBV, 350 million of whom are chronic carriers of the virus [1]. The course of disease can vary from unapparent self limiting to chronic active hepatitis which may lead to death after many years or, following contact with HBV, can lead to fulminant hepatitis [2]. Chronic carrier states occur in 5 to 10% of patients infected in adult life and 85 to 90% of those infected during infancy [3]. The outcome of infection depends upon many factors such as host immune status, age at infection, level of viral replication and probably the genetic variability of virus influencing the expression of viral antigens [4]. However, the impact of the natural variability of the virus on the clinical course of disease has become the focus of research only recently.

Prior to definition of genotypes, HBV strains were distinguished by serological analysis, based on the immunoreactivity of an antibody to a limited number of amino acids in the major surface antigen, HBsAg. HBV was formally classified into four different subtypes that were afterwards subdivided according to the antigenic determinant of HBsAg in adw (adw2 and adw4), ayw (ayw1, ayw2, ayw3 and ayw4), adr (adrq+, adrq-) and ayr [5]. All known subtypes contain the a-determinant [6], which is encoded between amino acid residues 124 and 147. The difference between the mutually exclusive subtype-specific determinants, d/y and w/r, is generated by amino acid exchanges from K to R at residues 122 and 160, respectively [7,8]. It has been shown that each of the known HBsAg subtypes may belong to either one or several genotypes; although in such cases, the genotype involved, rather than the subtype, is more likely to correlate with the geographical origin of the strain [9], which has been associated with anthropologic history [9,10].

Genotypically, HBV genomes have been classified into eight groups; designated A-H based on an intergroup divergence of 8% [11] or 4%, using the gene S sequence [12]. Genotype A is pandemic, but most prevalent in Northern Europe, North America and Central Africa. Isolates belonging to genotypes B and C have been observed in Southeast Asia and the Far East. Genotype D has a worldwide distribution, and predominates in the Mediterranean region. Genotypes E and F are prevalent in West Africa and in the Amerindian population, respectively [13,14]. Recently, genotype G has been identified in the USA and France [15], and genotype H has been found in Central America [16]. The genotypes and subtypes have been suggested to be useful clinical and epidemiological markers [17,18], since it is well known that the genotypes vary in geographical areas and correlate strongly with ethnicity [13,19].

Brazil is a federation of 26 states and one federal district occupying approximately 8,500,000 km^2 ^and divided broadly into five geographic regions: North, Northeast, Central-West, Southeast and South. In Brazil, wide variations in HBV infection prevalences have been reported, depending upon the geographical region [20,21]. Brazil, a country with a highly miscegenated population, exhibits an HBV genotype circulation pattern that is distinct from the distribution found in other Latin American countries, with genotypes A, D and F being the most prevalent among HBV carriers [22-24]. The distribution and characteristics of HBV genotypes in different regions of Brazil have not been fully studied. This study was carried out in Campinas, an industrialized urban area with approximately one million inhabitants, in São Paulo state, Southeast Brazil, where the estimated prevalence of HBsAg in blood donors is around 1.5% (low rate) [25]. The aims of this study were to identify HBV genotypes in HBV strains obtained from HBV-infected patients and to correlate them with clinical and histopathological features.

## Methods

### Patients

One hundred and thirty-nine consecutive serum samples from HBsAg-positive patients were studied (89 males, 50 females, mean age 42 ± 14.5, range 7 to 80 years, 117 Caucasian, 20 Afro and 2 Asian descendents). All patients were followed up by the Hepatitis Study Group of the Hospital das Clínicas da Faculdade de Ciências Médicas-UNICAMP, Campinas, São Paulo, Brazil, from 2005 to 2007. Inclusion criteria were HBsAg-positive patients with anti-HCV and anti-HIV negative markers. Serum samples were stored at -20°C and thawed immediately before use. Patients' records were consulted for data on gender, birth date, probable source of infection (use of intravenous drugs, transfusion, tattoos, healthcare professional or unknown), serological markers for HBV (HBeAg, Anti-HBeAg, Anti-HBcAg, anti-HBsAg) and liver biopsy results. The stage of fibrosis and degree of inflammatory activity were evaluated by the Metavir score system [26], which classifies fibrosis into five stages: F0 - no fibrosis, F1 - portal fibrosis without septa, F2 - portal fibrosis with few septa, F3 - portal fibrosis with numerous septa, without cirrhosis, F4 - cirrhosis. The degree of inflammatory activity was divided into: A0 - lack of histological activity, A1 - mild histological activity, A2 - moderate histological activity, A3 - severe histological activity. The Institutional Ethics Committee approved this study and informed consent was obtained from all subjects.

### HBV DNA Detection

Extraction and amplification of HBV-DNA were carried out by nested polymerase chain reaction (PCR) as described by Kaneko et al (1989), somewhat modified [27]. Selected primers corresponded to conserved regions among the different genotypes and surrounding regions that were heterogeneous among them, allowing the distinction of HBV genotypes. The selected region for amplification also covers the amino acid loop corresponding to *ad/y *and *w/r *specificities and mutations related to HBIg, anti-HBs monoclonal antibody and vaccine resistance. Selected primers were as follows: HBS1F 5'-GAG TCT AGA CTC GTG GTG GAC TTC-3' and HBS1R 5'-AAA TKG CAC TAG TAA ACT GAG CCA-3' and inner HBS2F 5'-CGT GGT GGA CTT CTC TCA ATT TTC-3' and HBS2R 5'-GCC ARG AGA AAC GGR CTG AGG CCC-3'). The primer positions in the HBV genome (strains HBV ADW; Genbank accession number V00866) were as follows: HBS1F (positions 244 to 267), HBS2F (positions 255 to 278), HBS2R (positions 648 to 671) and HBS1R (positions 668 to 691) [20]. Serum samples were subjected to two rounds of amplification with outer (HBS1F and HBS1R) and inner (HBS2F' and HBS2R) primers, sequentially. The amplification conditions for the two rounds of PCR were as follows: initial denaturation at 94°C for 20 s, followed by 30 cycles; 94°C for 20 s, 56°C for 20 s and 72°C for 30 s, with a final extension step at 72°C for 1 min, in a DNA Thermal Cycler 2400 (Perkin Elmer Cetus, Foster City, Ca, USA). The nested PCR product was a 417 bp fragment covering a region of S gene.

An aliquot of water was used as a negative control and serum from an HBV-positive individual, according to EIA, quantified by the Amplicor HBV Monitor test (Roche Diagnostic Systems, Branchburg, NJ) (1.0 × 10^2 ^copies/mL), was used as a positive control. Serial dilutions were made in an HBsAg low-titer performance panel (PHA 105; Boston Biomedica, Inc., Boston, Mass), from which the detection limit of this nested PCR was determined as being 10^2 ^copies/mL. Carryover contamination was prevented as described by KWOK & HIGUCHI [28]. The final amplification product was mixed with bromphenol blue, eletrophoresed in a 2% agarose gel, visualized by UV fluorescence after staining with ethidium bromide, and photographed with Polaroid film. Samples were considered positive if they yielded at least two positive results from three amplifications. Samples were considered negative when there were two negative results in two different reactions.

### Analysis of HBV Sequence from Different Genotypes

The products of nested PCR were subjected to cycle sequencing reactions, as described previously [29], by using the second-round primers and the ABI Prism Bigdye Terminator Ready Reaction kit (Applied Biosystems, Foster City, CA). After purification, samples were denatured and directly sequenced by automated cycle sequencing (ABI 377, Applied Biosystems, Foster City, CA). Revision of the nucleotide sequences was carried out using the BioEdit program version 5.0.6 [30]. Sequences were then submitted to the Blast program in order to check their similarity to other HBV strains deposited in GenBank. The results of sequencing were consistent with those of HBV isolates obtained from GenBank, corresponding to the known HBV genotypes. The genotypes were assigned to known groups using phylogenetics inference with known genotypes corresponding to eight HBV genotypes identified in humans and deposited in the Genbank: Genotype A (X75666, EF690536; subgenotype A1 = AF418672, subgenotype A2 = AB64314; subgenotype A3 (Acmr) = AB194951), B (X75660 ayw1; D00330 adw2; AB191369): C (D12980 adr; EU678472; AB24113.1); D (AB090270 ; X75668 ayw3; X59795 ayw2) E (X75657; AP007262; X75664) F (X75658; X69798 adw4; AY179735) G (AP007264; AB191378) H (AB191383; AB298362) and Genotype G/C recombinant (DQ078791).

### HBV DNA Quantification

Of the total number of samples (139), only seventy positive samples (17 HBeAg positive and 53 HBeAg negative) were submitted to HBV DNA quantification using the commercial Amplicor HBV Monitor assay (Roche Diagnostic Systems, Branchburg, NJ) with a threshold limit of 400 to 40,000,000 copies/ml

### Statistical Analysis

The student t-test and the Mann-Whitney test were used to analyze quantitative data. The χ^2 ^test with either the Yates correction or Fischer's exact test was used to analyze quantitative data and for comparing proportions. All calculated P values were two-tailed; a P value of less 0.05 was considered significant.

## Results

The patients' characteristics and the frequencies of the viral genotypes in the 139 patients are shown in Table 1. One hundred and seventeen (84.2%) of them were Caucasian descendents, 20 (14.4%) were African Brazilian descendents and 2 (1.4%) were Asian descendents. In this group, genotype A was the most prevalent (55%) followed by genotype D (38%), F (4%) and C (3%). The following subtypes were found, adw2 (48.2%), ayw (44.6%), adw4 (4.3%) and adr (2.9%). In genotype A, subtypes adw and ayw were detected. Genotypes C and F presented only subtypes adr and adw4, respectively. In genotype D, subtype ayw was the most frequent, with three patients bearing subtype adw.

**Table 1:** Characteristics and HBV genotypes frequencies among 139 HBV-infected patients

HBsAg Positive Patients		N = 139 (%)
Gender	M/F	89/50 (64/36)
Mean Age	42 ± 14.5	
Range of age (years)	7-80	
		
	Caucasian	117 (84.2)
Race	Asian	2 (1.4)
	African Brazilian	20 (14.4)
		
Risk factors	Uncertain	85 (61)
	Parenteral	28 (20)
	Sexual	15 (11)
	Vertical	11 (8)
		
Genotype	A	76 (55)
	C	4 (3)
	D	53 (38)
	F	6 (4)
		
Subtype	adr	4 (2.9)
	adw2	67 (48.2)
	adw4	6 (4.3)
	ayw	62 (44.6)
		
HBeAg	Positive	42 (30)
HBeAg	Negative	97 (70)
		
Inactive carrier	ALT<1.3 ULN	83 (60)
Chronic hepatitis	ALT>1.3 ULN	56 (40)
		
qALT medium	2.03	

ALT = alanine aminotransferase

ULN = unit limit normal

q = coefficient of ALT = U/ULN

Among the 76 patients infected by genotype A, 18.4% (14/76) of patients were African descendents, and of those infected by genotype D, 11.3 (6/53) were African descendents. Among the patients infected with genotype C, two were Asian descendents and two were Caucasian. Genotype F-infected patients were all Caucasians without notified indigenous origin.

The data analysis regarding risk factors showed that, in 28 (20%), the parenteral route was the most common risk factor while the sexual route was present in 15 (11%), and probable vertical transmission occurred in 11 (8%); although it is important to point out that in 85 (61%) patients, identification of the risk factor was not possible.

Fifty-six (40%) patients had chronic hepatitis (elevated ALT value), and 83 (60%) were probably inactive carriers (normal ALT value). The aminotransferase level was measured using qALT, which represents the value obtained by measuring samples of each patient and dividing by the normal unit limit (U/NUL). Thus, the qALT medium in this group was 2.03. In our study, 97 (70%) of patients were HBeAg negative and 42 (30%) HBeAg-positive (Table 2).

**Table 2:** Characteristics and genotypes among HBV-infected patients, according to HBeAg status (n = 139).

Characteristics		HBeAg positive (n = 42)	HBeAg negative (n = 97)	p-value
Mean age	(years)	37 ± 17.2	44 ± 12.7	0.0055
				
Gender	M	31 (73.8%)	58 (59.8%)	0.1139
	F	11 (26.2%)	39 (40.2%)	
				
Genotypes	A	25 (59.5%)	51 (52.6%)	0.1367*
	C	3 (7.1%)	1 (1.0%)	
	D	12 (28.6%)	41 (42.3%)	
	F	2 (4.8%)	4 (4.1%)	
				
Inactive carrier	(ALT<1.3 NUL)	18 (43%)	65 (67%)	0.0077
				
Chronic hepatitis	(ALT>1.3 NUL)	24 (57%)	32 (33%)	
				
qALT medium		2.5	1.8	0.0005
				
HBV-DNA	(copies/ml)	21.152.706	4.351.518	<0.0001

NUL = Normal Unit Limit

q ALT = U/NUL

*χ^2 ^= test

p value < 0.005 = significant

We also found a lower percentage of patients with chronic hepatitis in the HBeAg-negative group than in the HBeAg-positive group (P = 0.0077). The mean qALT was significantly higher in HBeAg-positive (2.5) than in HBeAg-negative patients (1.8) (P = 0.0005). HBeAg-positive patients were significantly younger than HBeAg-negative patients (37 ± 17.2 vs. 44 ± 12.7 years), but there was no difference in gender between the two groups (P = 0.1139). The mean HBV DNA level was significantly higher among HBeAg-positive patients (21.152.706 copies/ml), compared to HBeAg-negative patients (4.351.518 copies/ml) (P = < 0.0001).

Among the 65 patients who were submitted to the liver biopsies (Table 3), a mean stage of fibrosis of 2.4 was determined for genotype A, 1 for genotype C, 2 for genotype D and 3 for genotype F (P = 0.1148). The mean stage of fibrosis in genotype A patients (2.8) was found to be significantly higher than the mean stage of fibrosis in genotype D patients (2.0) (P = 0.0179). In addition, there was no significant difference between the grade of fibrosis when the different genotypes were compared according to the presence or absence of HBeAg (Table 3).

**Table 3:** Liver fibrosis among HBeAg positive and HBeAg negative patients, according to HBV genotype (n = 65).

	HBeAg positive (n = 29)	Fibrosis stage	HBeAg negative (n = 36)	Mean Fibrosis stage	P-value*
GENOTYPE **A**	n = 17	2.1	n = 15	2.8**	0.0834
GENOTYPE **C**	n = 2	1	-	-	-
GENOTYPE **D**	n = 8	2	n = 20	2	0.9364
GENOTYPE **F**	n = 2	3.5	n = 1	2	-

** P < 0.05 when genotypes A and D were compared.

## Discussion

HBV genotyping has been studied extensively around the world. We detected genotypes A, C, D and F in our group of Brazilian patients from São Paulo state, Southeast Brazil. Genotype A was the most common and was found in 55% of the patients. Sitnik et al. [20] found genotypes A, B, C, D, and F in various Brazilian regions, while in Rio de Janeiro, Southeast Brazil, only the genotypes A, D, and F were found [31]. These same genotypes have also been encountered in populations from other Brazilian states [32,33]. The population of Southeast Brazil contains many European and African descendents, which would explain the high prevalence of genotype A. We also encountered a relatively high rate of genotype D (38%), probably because São Paulo state received many immigrants from the Mediterranean region.

To our knowledge, this is the first time that genotype C has been detected in Brazilian patients that are not of Asian descent. Among our four genotype-C patients, two were non-Asiatic. Several studies carried out with Oriental Asians have shown that genotypes B and C are the most common in that area of the world. Patients infected with these genotypes have a poor prognosis, with more frequent progression to cirrhosis and hepatocellular carcinoma [34]. Genotype C infections are more severe than genotype B infections [35]. Other studies in China have reported that the higher rate of mutants of the basal core promoter (BCP) in patients with genotype C is associated with a greater pathogenicity of this genotype [35,36].

As a group, the most important predictor of elevated ALT and high HBV-DNA level was HBeAg status: HBeAg-positive patients were 1.5 times more likely to have elevated ALT and high HBV-DNA, compared with HBeAg-negative patients. The degree of observed difference in ALT and HBV-DNA levels between HBeAg-positive and HBeAg-negative patients is related to the age of the patients and the referral pattern.

Among our 139 patients, 56 (40%) had elevated ALT levels. Among the HBeAg positive patients, 57% showed high ALT levels and, among the HBeAg-negative patients, 33% had high ALT levels (P = 0.007). HBeAg-positive patients generally had higher virus levels when compared to HBeAg-negative patients. This was also observed in our study when we compared the viral load based on HBV-DNA, which was about 4.9 times higher among HBeAg positive patients, when compared with HBeAg negative patients.

The high degree of replication, which probably means more intense inflammation in the HBeAg-positive patients, has also been observed in other studies [37-39]. The mean ALT level in HBeAg-positive patients was 2.5, compared to 1.8 in HBeAg negative patients, which could be due to a higher degree of inflammation.

Among HBeAg-positive patients, we found 43% of inactive carriers, compared to 57% of patients with chronic hepatitis B (Table 2). Among 56 patients with chronic hepatitis B (HBsAg positive, HBV-DNA positive and increased ALT), 24 (43%) were HBeAg-positive and 32 (57%) were HBeAg-negative. This demonstrates the high percentage of cases of HBV patients that were negative for HBeAg in our population. In other studies, with different populations, considerable differences were also observed between the percentages of HBeAg-positive and HBeAg-negative patients thus the authors encountered a higher prevalence of HBeAg-negative patients, varying from 52.5% to 63.3% [39-41]. Similar percentages were also observed in another study in Brazil, in which 58% of the HBV patients were HBeAg negative [42]. About 30% of our patients were HBeAg positive and 70% were HBeAg negative. Probably the majority of the HBV positive patients in our region had been infected for a long time and had developed mutations in the pre-core region (HBeAg negative/anti-HBeAg positive).

When we examined the intensity of liver lesions, we did not observe significant differences between the two groups, although the HBeAg negative patients had usually been infected for longer. The fibrosis stage was significantly greater among the HBeAg negative patients infected by genotype A, when compared to patients infected by genotype D. We believe that this occurred because approximately 18.3% of our genotype-A-infected patients were African descendents

## Conclusion

The genotypes encountered in our HBV-infected patients were apparently a consequence of the types of immigration that occurred in our region, where immigrants predominate from European and African countries. In our HBV-infected patients, HBeAg-negative was in the majority, which is associated with the time of infection and with the presence of HBV core mutants. The viral load in HBeAg-positive-patients was 5 times higher than HBeAg-negative individuals (higher grade of replication). The genotype A patients showed more significant fibrosis than the genotypes D and F in our population.

## Competing interests

The authors declare that they have no competing interests.

## Authors' contributions

PAT carried out laboratory analyses in the molecular genetic studies and participated in the analysis of the study. NSL, participated in the design of the study, performed the statistical analysis, conceived the study, and made substantial contributions to acquisition, analysis, and interpretation. AGV, ESLG made substantial contributions to acquisition, analysis, and interpretation of data. AF performed liver biopsies and participated in the design of the study. VCF carried out the molecular genetic studies and drafted the manuscript. FLG Jr, was involved in drafting the manuscript, revising it critically for important intellectual content and coordination. All authors read and approved the final manuscript.

## Pre-publication history

The pre-publication history for this paper can be accessed here:

http://www.biomedcentral.com/1471-2334/9/149/prepub
